# On Mesmerism, Improperly Denominated Animal Magnetism

**Published:** 1829-03

**Authors:** Richard Chenevix


### ANIMAL MAGNETISM.

*On Mesmerism, improperly denominated Animal Magnetism.* By Richard Chenevix, Esq. F.R. and E.S. M.R.I.A. &c.†

Animal magnetism is true. In the whole domain of human acquirements, no art or science rests upon experiments more numerous, more positive, or more easily ascertained. As this assertion is in direct contradiction to the

† We are informed that Mr. Chenevix intends paying us a visit in London and that he is prepared to convince the most sceptical that "mesmerism" is not the system of juggling imposition which by many it has been believed to be. For our own parts, we candidly confess we are yet to be converted. Our opinions upon this subject are fully stated in the November Number of 1826. We hope Mr C. will give us an opportunity of witnessing the effects of animal magnetism.—Editors.

vast majority of current prejudices, it is just to state the grounds upon which it is made.

In former times, whenever animal magnetism was mentioned, I joined the general tribe of scoffers; and so much was I convinced of its absurdity, that, being at Rotterdam in 1797, I laughed to scorn a proposal made to me by an English resident there, to witness some experiments in which he was then engaged. His assertion was, that a somnambulist of his should, in her sleep, without any signal from him, leave her chair, and seat herself on any other chair which he should mentally designate. The respectability and general understanding of this person left no mode of accounting for so extraordinary an illusion, but to suppose him labouring under monomania.

In 1803 and 1804, while travelling in Germany, I heard many very enlightened men of the universities talk of animal magnetism, nearly with the same certainty as of mineral magnetism; but their credulity I set down to the account of German mysticity, and thought it not incongruous that the nation which took its philosophy from Kant, and Tiehte, and Schelling, should believe that certain motions of the hands could, by the will of the mover, transmit an influence to the person acted upon, which should produce the wonders related of animal magnetism. I remained an unbeliever.

In 1816, some persons of my acquaintance proposed to take me to the house of a lady in Paris, whose daughter was an artificial somnambulist, and, in the terms of the art, lucid. I went to laugh: I came away convinced.

To suspect any thing like a trick in the parties concerned was impossible. They were of the highest respectability and distinction, and some of them I had known for many years. The magnetiser was, indeed, in the frivolous French metropolis, called a charlatan, which made me suppose that he was not so; and the event proved that I was right. He was, indeed, poor; he exercised his art for money; he gave public lectures at three francs a ticket. Many young physicians have as fair a claim to the title as he had. But, from the hour above alluded to till the period of his death, I remained acquainted with the Abbé Faria, and never knew a man to whom the epithet impostor was less applicable.

No sooner had the Abbé Faria begun to operate than the countenance of the young lady changed, and in two seconds she was fast asleep, having manifested symptoms which could not be counterfeited. The sitting lasted about two hours, and produced results which, though I still remained a sceptic upon some of the most wonderful phenomena, entirely convinced me of the existence of a mesmeric influence, and of an extraordinary agency which one person can, by his will, exercise upon another. The Abbé Faria offered every means to dispel my remaining doubts, and gave me all necessary instructions to obtain total conviction from experiments of my own. I most zealously attended his labours, public and private, and derived complete satisfaction upon every point relating to mesmerism; even upon those which appear supernatural. Many of the experiments I repeated, not only upon persons whom I met at his house, but upon others totally unacquainted with him or with his studies, and was ultimately compelled to adopt the absolute and unqualified conclusion announced above: "Mesmerism is true." Other occupations, however, prevented me from continuing the subject, and I had only casual opportunities for exercising the art, until accident called back my whole attention to its truth and importance.

Witness of some of the wonders which have lately been the subject of discussion in the French Academy of Medicine,* and surprised at the pusillanimity of that body, which cannot deny, yet has not manliness enough to avow, the facts which one half of its members declare they have witnessed, I resolved, with all due humility, yet not shrinking from the task, to devote some time to the collection of facts, and to offer the results to a much more enlightened public than that to which the art is compelled to appeal in France.

My first opportunity for renewing my practice was in May 1828, when, happening to be on a visit to Ireland, I inquired for some patient among the peasantry, no matter what the disorder.

Jane Hurly, an epileptic woman, aged thirty-four, was produced; and as, but a short time before, she had been seen in a fit by a person of the family at whose house I was residing, there could be no doubt of the reality of the distemper. Besides, she had lately fallen into the fire in a paroxysm, and most dreadfully burnt her leg. She had been six years epileptic; had a strong tendency to paralysis of the left leg and thigh; was subject, almost daily, to spasmodic contractions in her hands and feet, accompanied by racking pain, and which sometimes lasted twelve hours

* Expériences publiques sur le Magnetisme Animal faites à l'Hôtel-Dieu de Paris, par J. Dupotel. Also the case of Paul, in the Hermes; by Dr. Foissac.

Délibérations de l'Académie de Médecine sur le Magnetisme Animal. Rapport fait par M. Husson.

or more; had occasional absences of mind and loss of memory; never slept more than a couple of hours at once, and that but rarely; was constantly thirsty; her appetite was bad. She was eight months advanced in her sixth pregnancy, and it was after her first confinement that she had her first attack.

Tuesday, May 23d.—Mesmerised her for forty-five minutes: no sleep, but a little drowsiness.

24th.—Night better than usual; no spasms in hands or feet. Mesmerised her again forty-five minutes: no sleep. Gave her mesmerised water to drink.

25th.—Felt heavy and drowsy ever since yesterday. Mesmerised her again forty-five minutes: no sleep.

On the 26th, I did not mesmerise her.

27th.—The day before yesterday, she had a return of the spasmodic contractions of the hands and feet, but they lasted only two hours. This day, after mesmerising her for nine minutes, she fell into mesmeric sleep. She feels herself stronger and better than when the treatment was begun.

On the 28th, I did not see her.

29th.—She fell asleep in three minutes, but awoke as soon as spoken to. Yesterday she had a second return of the spasms, but only in one foot, and for a few minutes. The use of mesmerised water, begun on the 24th, had entirely assuaged the thirst, which used to be habitual and intense. Dr. M'Kay, whom I shall presently mention, was present at this sitting.

30th May, 1st and 3d of June.—Fell into complete mesmeric sleep after two minutes' mesmerising. Her health is improving rapidly.

5th June.—The person at whose house I was visiting being desirous of seeing some effects of mesmerism, I put the patient to sleep in his presence in six minutes, by my will alone, and without any visible manifestation of it.

7th.—In the presence of the same person, I mesmerised the patient through the door, and at the distance of fifteen feet; she not knowing that I was acting upon her, but supposing that I was absent; and in fourteen minutes she was in complete mesmeric sleep.

10th.—Being absent, I did not see this woman for two days. In the interval she had a severe spasm in her left leg and thigh for six hours, followed by cold and numbness in those limbs. This day I put her to sleep in half a minute, and mesmerised the part affected. In forty minutes I wakened her: the pain was gone, and the limbs had recovered their natural strength and heat. This was the last return of these symptoms. By this time she had completely recovered her sleep, not only at night, but was frequently obliged to lie down in the day, after quitting me. She now slept ten or twelve hours in the twenty-four, and one day sixteen hours. She continued rapidly to improve in health, and her appearance was so much changed that her neighbours, who knew nothing of the treatment, were struck at the alteration. The operations were continued until June 20th, when her pregnancy made her unable to come out; and on June 28th she was delivered.

July 6th.—I went to her house, and found her up and well, with the exception of rheumatic pains in her left shoulder, for which I mesmerised her. She soon felt them descending to her elbow, and thence to her wrist, and in less than ten minutes was perfectly relieved.

17th.—She came to thank me for her entire recovery; and, to prove it to myself, I mesmerised her during thirty minutes, with the strongest will to put her to sleep; but, though formerly she fell into mesmeric sleep in half a minute, I could not now produce the slightest effect upon her.

I repeated the same experiment the next day, but with no effect. I did not then see her until September 17th, when I again attempted, but in vain, to produce mesmeric sleep.

It would be childish to attribute the cure of this woman to her pregnancy or her confinement. From the very first day she was mesmerised, the symptoms were alleviated, and decreased regularly as the treatment advanced. In less than a week, thirst, insomnia, shiverings, and pains, to which she had been subject for six years, ceased; the paralytic tendency diminished; and the spasmodic contractions were entirely removed after the twelfth day of mesmerising. Before her confinement, her health was completely restored, and she has not had a return of epilepsy for nine months, though the attacks were formerly very frequent. If this case does not offer a fair affiliation of cause and effect, there is no truth in deducing the cure of ague from the administration of bark.

Although none of the extraordinary phenomena of lucidity occurred; although this patient awoke the instant she was spoken to; her cure is interesting, as being completed so rapidly. Twenty-one sittings sufficed; and after them, and the cessation of all former symptoms, I could not produce any sensible effect upon her. Even at the period when she used to be most affected, the touch of my finger, so slight as to be almost imperceptible to myself, roused her from her state of mesmerism, and with a sensation which she described as like the prick of a pin. I have known some educated persons, who experienced a similar sensation, compare it to an electric spark.

Epilepsy is one of the diseases where the medical art is the most in default. It is also one where mesmerism effects the slowest cure. I have known a case in which this powerful agent was employed daily, for an hour each time, during seventeen months, and in which, though the symptoms had begun to yield almost at the first sitting, the cure was not complete before that period. Its frequency among the lower classes is extreme; for in six months I saw thirty-seven cases, thirteen of which I treated myself, by no other means than mesmerism. In three of these cases I was completely successful; in eight more I procured immense relief; two only were failures. Four of the eight are still under treatment by me, and the remaining four are treated by a relation of each respective patient.

Judith Doonah, a laundress, was afflicted with violent pains in her head, which returned periodically every sixth day, and the precise nature of which was not entirely ascertained by physicians. In the opinion of some they were rheumatic; according to others, they partook of the nature of tic douloureux. They came at the end of a bad ophthalmia, and continued long after that disease had subsided. The right temple, cheek, eye, and shoulder, were the parts particularly affected.

June 23d.—I mesmerised her for the first time, and during a paroxysm. In a very few minutes she found relief. I repeated the operation thirteen times, but, being occupied with other patients, I could not sufficiently attend to her. I gave her, however, mesmerised water to bathe the part affected, and a piece of mesmerised glass to wear upon the temple. From June 29 till July 20, she had no return of the pains, but on the latter day she had a slight attack. Shortly after this I left the country where she was, and did not see her for two months. The pains had returned, though not quite so frequently. As I had not time to mesmerise her constantly, I recommenced the mesmerised water and glass; and in six weeks she was completely cured, without the use of any other remedy.

Between May 23d, 1828, and January 20th, 1829, I tried the effects of mesmerism upon 164 persons, of whom 98 manifested undeniable effects; some in one minute, some not till the operation had been repeated several times. There was hardly an instance where disease existed, that relief was not procured; and many of the patients offered phenomena as extraordinary as any recounted in Germany or France. The space allotted to this communication does not allow a minute relation of them, and I must confine myself to turning the public mind to this most wonderful agent, which, like all that is new, has been assailed by ignorance, by prejudice, and by ridicule, yet which is as true as gravitation or affinity. While prosecuting these experiments, I had the good fortune to meet with many benevolent and zealous persons, not of the faculty, who have made trial of the art with entire success, having hardly ever failed to procure relief for their fellow-creatures, at the same time that they produced phenomena which highly surprised and gratified them. I can at this moment count at least fifty persons who have become converts and practitioners in consequence of what they had heard or seen, directly or indirectly, by my means; and who have assuaged the pains, if not cured the diseases, of some hundreds of suffering individuals, without the aid of medicine. To this list must be added three enlightened practitioners, Drs. M'Kay, Cotter, and Peacock, all of them physicians to public establishments in the neighbourhood of the place where the experiments were made. The former kindly lent his assistance upon all occasions, to determine the nature of the disease, the progress of the cure, &c. and witnessed many wonderful phenomena, of which he is ready to testify the truth to all who may require it. The protracted scepticism of the second led to the following trial:

On Thursday, October 2d, I requested Dr. Cotter to be present at some mesmeric experiments. He saw two epileptic patients put to sleep in about half a minute each. One of them, while under the mesmeric influence, had a slight fit, which, by increasing the action, I arrested instantaneously. The other he saw me strike motionless, by my will alone, as she walked across the room, and set at liberty in an instant by the same agency. To these facts he, as well as two other gentlemen present, could not refuse their assent; but still a suspicion of connivance and trick might lurk in his mind. I requested him to bring me any five patients of his own, whom he was sure I never could have known or heard of, and to hang his conviction upon this test, that I would, in half an hour, produce effects upon one of these five, which should convince him of the existence of the mesmeric influence.

On Saturday, October 4th, he came with a female patient, whom he had been treating for dyspepsia, costiveness, and headach, during four years. Her usual aperient dose was thirty grains of jalap with ten of calomel. I never saw her till that day, and only in the presence of Dr. Cotter. She had no idea of what was to be done to her, and was at the moment suffering with severe headach. In three minutes' mesmerising she said her headach was better; in five minutes she said it was quite well. In eight minutes she was in one of the soundest mesmeric sleeps I have witnessed, and continued so for thirty-five minutes, when I awoke her.

While she was asleep, Dr. Cotter said to me, *in Latin*, that her bowels were at that moment particularly bound.

I directed my attention to procuring an evacuation, passing my hands before the abdomen, without, however, touching it, or approaching nearer to it than three or four inches. In less than an hour after she had left the house, she had three evacuations, and for some days her head was considerably relieved. This patient lived at too great a distance for us to continue the treatment; but the following note from Dr. Cotter, relating to another patient, was this moment brought to me:

"Within this week I have witnessed the effects of mesmerism in the case of Miss P., aged fourteen. She had long been subject to an irregular pain in the left side, over the kidney, accompanied, in its attacks, with a sinking, as described to me, or a tendency to faint. Having long tried medicine without any permanent good, I was desirous to leave off a habit so injurious to a growing subject. I had recourse to mesmerism, without describing to her what I was going to try. Four minutes produced a complete state of somnolency. I have performed it upon this subject but three times, and she has had no return whatever of the pain in her side; neither has there been occasion to exhibit aperients, for which, previously, there was a continual necessity."

That any person, whether a believer or not, can produce mesmeric phenomena, may be learned from the following fact: A lady of a very robust frame, and a very energetic will, had heard and read much upon mesmerism, but was not convinced. Three patients, whom I had never seen before, were waiting for me. I proposed that she should try whichsoever of them she pleased. The most unhealthy female was selected. In two minutes the patient's head dropped, but she started up immediately; in less than four minutes, however, she was fast asleep. Here neither the mesmeriser nor the mesmerisee had the slightest conviction upon the subject, yet the experiment succeeded as completely as with the most habituated professor.

Neither previous knowledge nor education is necessary for the development of this precious faculty, in those who heartily wish to exercise it.

December 2d, 1828, Catherine Nicolson, a woman of the very lowest class of Irish peasantry, brought me, on her back, her daughter, aged nine, dreadfully afflicted with scrofula. She had seven sores near her knee. I instructed this woman how to mesmerise her child.

12th.—She came to tell me that some of the ulcers were disposed to heal, and that a splinter of bone had come out of one of them.

January 2d, 1829.—The girl can stand alone, and walk with a crutch. Two more splinters of bone have come away, and the ulcer which voided them is very much inflamed; the others being better.

19th.—Three more splinters of bone have come away, one of them three fourths of an inch long, and as thick as a crow's quill.

Another girl, Bridget Hedouin, is nearly cured of epilepsy, by her father, an ignorant peasant, whom I taught to mesmerise her; the attacks being reduced to one fourth in frequency, duration, and intensity, since December 3d, 1828, when the treatment was commenced.

Both the above treatments are now in progress.

I have at this moment eleven cases of different diseases, in which a friend or relation is the operator, and nine of which are proceeding with the most extraordinary success.

I could here enumerate near two hundred examples, but I am fully aware that, in the present condition of the science, these things must be seen to be credited. I shall not, then, attempt to argue or convince my readers, but to implore them to try the experiment themselves. Every one can mesmerise, though not all with equal effect, and practice increases the power: but it is not every one who is susceptible of a sensible influence from this agency; and somnambulism is generally estimated not to occur more frequently than in one case out of five, and not one in twenty-five patients becomes lucid.

I was myself an unbeliever until I was undeceived by my own experiments: but, had I sooner taken this plain and rational road to knowledge, instead of thinking all men mad who trusted to their eyes that told them truths, which to me seemed more marvellous than all the other wonders of creation, I should, many years since, have possessed the conviction which I now enjoy, and not bewail that, in 1797, my presumptuous ignorance had shut in my own face the door of a science more directly interesting to man than all that chemistry and astronomy can teach. Nine tenths who may read will laugh at this, as I did at my friend at Rotterdam. Let them do so; but, while they laugh, let them learn, and not, thirty years afterwards, have to lament that so short a remnant of life is left to them to enjoy this new and most valuable secret of nature.

In Germany this science has long been practised; and in Berlin an hospital was established in 1815, in which no medicine but mesmerism, and the prescriptions of lucid somnambulists, was used. Hüfeland, once a scoffer, but converted; Hüfeland, in himself a host, was at the head of this hospital, and fifteen volumes of mesmeric cases have been published. Even in Holland, last year, I found the truth of mesmerism hardly doubted; and I met with some patients at Aix-la-Chapelle who had reaped benefit from it. In France it has been believed, and reviled, and believed again, and has followed all the vicissitudes of fashion. In England it has never risen above the level of quacks, and there that level is low indeed. Its fate in these countries was exactly analogous to the characters of the respective nations. The Germans were attracted towards it by their love of mysticity, and hailed it on account of its marvellousness. This once, however, the spirit which has so often been prejudicial to the German mind, has led to truth, while other nations turned aside from the path of knowledge. In France, where words and jargon are more valuable than facts, it has been treated as a matter of opinion, not of experiment. Though all the phenomena have been produced over and over again, yet, as these phenomena are not phrases, the Academy of Medicine thinks it can argue down somnambulism, and talk lucidity out of existence. The repugnance of English minds to the supernatural in science has prevented them even from bestowing a thought upon the subject; but let a few authentic results be known, and, in this seat of powerful understanding, it will make more rapid strides in one year, and without the assistance of governments or academies, than it has done since 1784, when aided by mysticism or garrulity. Nothing can be more fair and candid than the language spoken by the partizans of mesmerism in all countries. Instead of calling themselves gifted beings in whose hands alone the power resides, they say to unbelievers, "Come, and see," and then "Go, and try."

Natural somnambulism has in all ages been so often seen, and so well authenticated, that to deny it would be absurd. Now what is artificial somnambulism, and what lucidity, but the same state as the former, produced and regulated by certain principles which all men can command. Wonderful, indeed, it may appear; but what makes any thing wonderful to us, if not our ignorance. Since the world began, men have been wondering at every thing, till habit tamed their minds upon it. In my remembrance, they have wondered at hydrogen and oxygen; at a dead frog jumping between two slips of metal; at gas-lights, and steam-boats; and now they wonder at all who wonder at those familiar themes. They would pity the wretch who would not instantly believe that a stone falls, and a balloon rises, by the same impulse; or that the taste which his tongue perceives when placed between a piece of silver and a piece of zinc, has the same origin as the thunder which strikes his soul with awe. Every thing in creation is wonderful, or nothing is so; but the last known truth always appears the most miraculous to unreflecting minds.

Much is to be apprehended from enthusiasm in this subject. Mesmerism is beneficial in all diseases, but it does not cure all cases of all diseases. It acts with equal success upon epilepsy, fever, rheumatism, diseased liver, diseased lungs, gout, scrofula, &c. Where bark would kill, where bark would cure, this agent may be alike applied. It is the mightiest of therapeutic aids, but it is not omnipotent. It is the most beneficent, too; but, in the hands of persons disposed to make a bad use of the ascendancy which it sometimes gives over the patient's mind, it might become the most dangerous. Let honest men, then, get possession of it, that they may be able to cope with knaves.

Neither is it pathologically or physiologically that this agent must principally be considered. Its psychological importance is far above the part which it can play in the art of healing. When a human being can, by the operation of another human being, see without his eyes, taste without his tongue, hear without his ears, and obtain complete insight into things of which, in his waking state, he had no knowledge, the condition of his mind in that moment is worth investigating. Yet these things are true: they are familiar to mesmerisers, and I myself have witnessed them full twenty times. They are not, however, every-day occurrences; and the novice practitioner must not be disheartened if he does not meet with them immediately: he must be content to produce, by his first efforts, some of the simplest phenomena. Let him persevere, and he will see the wonders which other mesmerisers have seen, and add new knowledge to our present stores.

However wonderful mesmerism may appear, one thing relating to it is still more wonderful, viz. that its truth has even been questioned; since it is in the power of every one, without previous knowledge, study, or acquirements, to obtain conviction at least in a week, perhaps in a few seconds. The truth which Pythagoras told of the earth's motion, reviled in its day, was reproduced by Copernicus two thousand years afterwards, and was again reviled; but this truth required all the mind of a Newton for its demonstration. Mesmerism, which the simplest motion of the hands, directed by the will, can prove, perhaps, instantaneously, has been discredited ever since Mesmer first revived it; and, before his time, was often believed, and as often forgotten. To me (and before many years the opinion must be universal,) the most extraordinary event in the whole history of the human science, is that mesmerism ever could be doubted. Go to p. 491.

---

### HOSPITAL REPORTS,

